# Comparative phylogeography of parasitic *Laelaps* mites contribute new insights into the specialist-generalist variation hypothesis (SGVH)

**DOI:** 10.1186/s12862-018-1245-7

**Published:** 2018-09-03

**Authors:** Conrad A. Matthee, Adriaan Engelbrecht, Sonja Matthee

**Affiliations:** 1 Evolutionary Genomics Group, Department of Botany and Zoology, Stellenbosch University, Stellenbosch, Western Cape Province South Africa; 2 Department of Conservation Ecology and Entomology, Stellenbosch University, Stellenbosch, Western Cape Province South Africa; 3 Department of Biodiversity and Conservation Biology, University of the Western Cape, Cape Town, Western Cape Province South Africa

**Keywords:** Specialist-generalist variation hypothesis, *Laelaps*, Phylogeography, Parasite evolution, Southern Africa, Dispersal

## Abstract

**Background:**

The specialist-generalist variation hypothesis (SGVH) in parasites suggests that, due to patchiness in habitat (host availability), specialist species will show more subdivided population structure when compared to generalist species. In addition, since specialist species are more prone to local stochastic extinction events with their hosts, they will show lower levels of intraspecific genetic diversity when compared to more generalist.

**Results:**

To test the wider applicability of the SGVH we compared 337 cytochrome oxidase I mitochondrial DNA and 268 nuclear tropomyosin DNA sequenced fragments derived from two co-distributed *Laelaps* mite species and compared the data to 294 COI mtDNA sequences derived from the respective hosts *Rhabdomys dilectus*, *R. bechuanae, Mastomys coucha* and *M. natalensis.* In support of the SGVH, the generalist *L. muricola* was characterized by a high mtDNA haplotypic diversity of 0.97 (±0.00) and a low level of population differentiation (mtDNA F_st_ = 0.56, *p* < 0.05; nuDNA F_st_ = 0.33, *P* < 0.05) while the specialist *L. giganteus* was overall characterized by a lower haplotypic diversity of 0.77 (±0.03) and comparatively higher levels of population differentiation (mtDNA F_st_ = 0.87, *P* < 0.05; nuDNA F_st_ = 0.48, *P* < 0.05). When the two specialist *L. giganteus* lineages, which occur on two different *Rhabdomys* species, are respectively compared to the generalist parasite, *L. muricola*, the SGVH is not fully supported. One of the specialist *L. giganteus* species occurring on *R. dilectus* shows similar low levels of population differentiation (mtDNA F_st_ = 0.53, *P* < 0.05; nuDNA F_st_ = 0.12, *P* < 0.05) than that found for the generalist *L. muricola.* This finding can be correlated to differences in host dispersal: *R. bechuanae* populations are characterized by a differentiated mtDNA F_st_ of 0.79 (*P* < 0.05) while *R. dilectus* populations are less structured with a mtDNA F_st_ = 0.18 (*P* < 0.05).

**Conclusions:**

These findings suggest that in ectoparasites, host specificity and the vagility of the host are both important drivers for parasite dispersal. It is proposed that the SGHV hypothesis should also incorporate reference to host dispersal since in our case only the specialist species *who occur on less mobile hosts* showed more subdivided population structure when compared to generalist species.

**Electronic supplementary material:**

The online version of this article (10.1186/s12862-018-1245-7) contains supplementary material, which is available to authorized users.

## Background

Comparative phylogeographic studies on parasites and their hosts are important to address ecological, evolutionary, and applied questions in parasitology [1]. Amongst others, it can facilitate the detection of cryptic lineages [2–7], aid in predicting the spread of diseases [8, 9] and can also provide insights into the mechanisms that play a role in parasite dispersal and gene flow [10–13]. The complexity and diversity of parasite systems, however, render accurate predictions on the factors responsible for parasite dispersal problematic. For example, host dispersal is not consistently correlated with parasite movement [14], parasites with broad host ranges can be highly structured due to biogeographic influences [15], and even obligate host-specific parasites do not necessarily show significant co-evolutionary patterns [5]. Through concerted efforts, however, some generalizations emerge such as the specialist-generalist variation hypothesis (SGVH) as proposed by Li et al. [16]. In short, this hypothesis proposed that since the habitat of specialist parasite species are patchier due to host availability, especially when compared to generalist species, specialist will show a more subdivided population structure [16]. In addition, specialist species are more prone to local stochastic extinction events (together with their hosts) than their more generalist counterparts, and this will result in lower levels of genetic diversity in specialist when compared to more generalist parasites [16].

Individual components of the SGVH are indeed reasonably well supported in terrestrial systems where generalist species show lower levels of population differentiation when compared to more specialized species [17, 18] and specialist also show significantly less genetic variation when compared to generalist [19, 20]. In the marine environment, however, the generalization of the SGVH hypothesis have been questioned due to the general lack of population structure among both specialist and generalist [21].

To test the paradigms associated with the SGVH we compare the genetic geographic structures of two evolutionary closely related nest bound ecotoparasite mite species, *Laelaps gigantues* and *L. muricola* [6, 13]*.* The two parasite species have overlapping distributions in southern Africa and it seems reasonable to suggest that samples taken from the same geographic locality will be broadly subjected to similar abiotic influences derived from the external environment. Since the two species are also morphologically similar [6, 22] they most likely have the same intrinsic abilities to disperse across the landscape. Both *Laelaps* species occur for short periods on the hosts for feeding, have low prevalence’s on their hosts [6, 23] and are characterized by female sex bias dispersal [18, 24]. The most obvious variables that can influence neutral genetic diversity and population structure in these taxa are linked to their individual level of host specificity [6] and the differential intrinsic abilities of the various host species to move across the landscape [15, 25, 26].

*Laelaps giganteus* is a host specialist that is found exclusively on the four striped mouse genus *Rhabdomys* [6]. The mite shows a significant signal of co-divergence [13] with the four *Rhabdomys* species recognized in the region [26], and each *Rhabdomys* species harbors their own unique *L. giganteus* lineage [13]. The host species are geographically differentiated from each other and only co-occur in very narrow contact zones [26, 27]. The individual *Rhabdomys* species, however, differ in their phylogeographic structure (genetic connectivity among geographic populations). For example, the solitary *R. dilectus* that occurs on the eastern area of southern Africa has haplotype sharing throughout the region (indicative of higher dispersal capabilities) while the arid adapted *R. bechuanae* has strong intraspecific population differentiation among sampling sites, indicative of lower dispersal among sampling sites [11].

*Laelaps muricola,* is a host generalist and has been recorded from the geographically co-occurring Southern multimammate mouse (*Mastomys coucha*), Natal multimammate mouse (*Mastomys natalensis*), and the Namaqua rock mouse (*Micaelamys namaquensis*). The generalist nature of *L. muricola* is confirmed by the absence of strong host association at the genetic level [6], suggesting that the parasites on the different host are not structured by host species. The dispersal capabilities of the various hosts can be inferred from previous phylogeographic investigations [15, 25]. In the case of *L. muricola,* both *Mastomys* host species show recent expansion events and extensive haplotype sharing throughout their southern African range [15], implying that they have a high dispersal capability. The third host of this parasite, *M. namaquensis,* show a much larger degree of population differentiation among sampling localities [25], suggesting several intraspecific barriers to dispersal throughout the region. Irrespective of the strong structure observed in the latter, gene flow patterns of parasites that use multiple hosts as part of their life cycle are predicted to be largely influenced by the vagility of their most mobile host [28–30]. In our case, gene flow in *L. muricola* should largely overlap with the “panmictic” pattern obtained in *Mastomys* species [15].

The objective of the present study is to use three closely related mite lineages (one being a host generalist and two being host specialist on two different *Rhabdomys* species), to test for congruence with the SGVH hypothesis as proposed by Li et al. [16]. The strength of our comparative approach lies in the fact that the parasites used in this study overlap in range (keeping environmental conditions constant), they have very similar life history characteristics, and published data on host dispersal and evolutionary history are available [5, 15, 25, 26]. It is proposed that this study will provide more direct insights into the effects of host specialization versus host movement on the genetic diversity and population structure of nest bound ectoparasites.

## Methods

### Taxon sampling

**Fig. 1:**
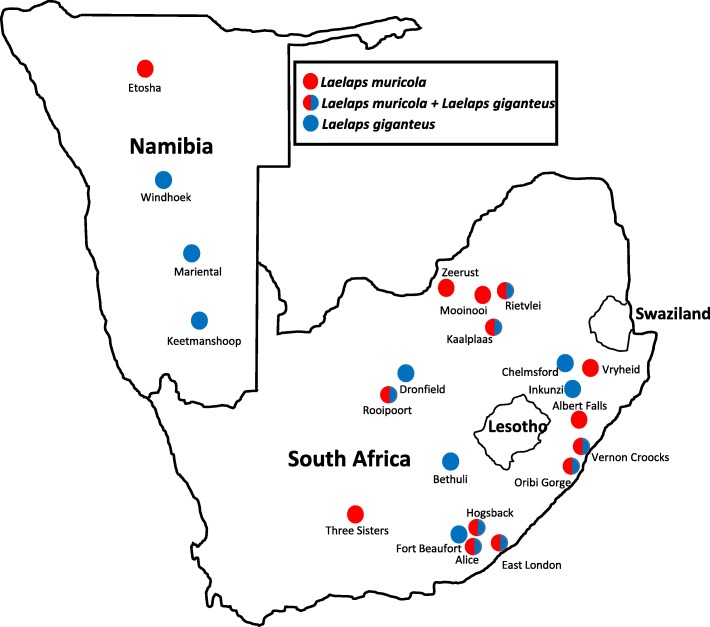
Southern African sampling localties of *Laelaps muricola* and *Laelaps giganteus* used in this study. Locality names correspond to those in Table 1

*Laelaps muricola* specimens were obtained from three different rodent hosts collected at 14 localities across southern Africa (Table 1; Fig. 1). Contrary to the taxonomic literature that indicates *L. muricola* to also occur on *Rhabdomys* [31], none of the *Rhabdomys* specimens included herein or elsewhere [6, 13, 23, 32] harboured any *L. muricola* specimens. Instead only *L. giganteus* was found on *Rhabdomys* [also see 6, 23, 32]. The *L. giganteus* data used herein were obtained from a previously published study [13]. To ensure geographic overlap between the two *Laelaps* species, only the parasites sampled from the geographically separated *R. bechuanae* and *R. dilectus* were included (16 localities in total; Table 1; Fig. 1). To be able to make comparisons based on host vagility, COI mtDNA data of *R. dilectus* (43 individuals from 11 localities; Additional file 1: Table S1) and *R. bechuanae* (50 individuals from 5 localities; Additional file 1: Table S1) together with similar data from the most mobile hosts of *L. muricola, M. natalensis* (106 individuals from 13 localities; Additional file 1: Table S1) and *M. coucha* (91 individuals from 14 localities; Additional file 1: Table S1) were downloaded from Genbank [13, 15, 26] or newly sequenced following procedures outlined in [26].

**Table 1:** Collection localities, host species, total number of individuals per host species for each gene fragment and Genbank Accession numbers are given in brackets

Locality	Hosts	COI	TropoM
*L. muricola*			
Rooipoort 28°38′27.9”S 24°16′45.9″E	*M. namaquensis*	9 (KU166723..31)	17 (MF412010..18)
Albert Falls 29°25′36.3”S 30°25′38.8″E	*M. natalensis*	9 (KU166736..44)	13 (MF412000..09)
Vryheid 27°48′00.1”S 30°45′43.2″E	*M. natalensis*	4 (KU166732..35)	*
Oribi Gorge 30°41′29.2”S 30°17′33.2″E	*M. natalensis*	5 (KU166683..87)	1 (MF412019)
Vernon Crookes 30°16′27.0”S 30°35′37.9″E	*M. natalensis*	4 (KU166679..82)	4 (MF412020..24)
Hogsback 32°35′56.4”S 26°56′05.7″E	*M. natalensis*	19 (KU166760..78)	*
Alice 32°48′55.2”S 26°50′21.5″E	*Mastomys sp.*	(7) (8) (KU166745..59)	1 (MF419355)
East London 33°00′33.0”S 27°51′04.7″E	*M. namaquensis*	5 (KU166673..78)	*
Mooinooi 25°44′48.9”S 27°32′58.6″E	*M. coucha*	12 (KU166709..20)	8 (MF419368..75)
Zeerust 25°31′57.4”S 26°03′03.4″E	*M. coucha*	2 (KU166707..08)	*
Rietvlei 25°52′20.7”S 28°16′38.6″E	*M. coucha*	8 (KU166694..701)	*
Kaalplaas 25°35′28.0”S 28°09′26.4″E	*M. coucha*	11 (KU166702..06; KU166688..93)	6 (MF419401..06)
Etosha Pan 19°01′36.2”S 16°23′54.3″E	*M. namaquensis*	3 (KU166779..81)	*
*L. giganteus*			
Oribi Gorge 30°41′29.2”S 30°17′33.2″E	*R. dilectus*	11 (KU166634..44)	1 (MF419641..42)
Chelmsford 27°57′19.0”S 29°55′51.6″E	*R. dilectus*	19 (KU166534..54)	16 (MF419523..54)
Vernon Crookes 30°16′26.6”S 30°35′33.6″E	*R. dilectus*	14 (KU166659..72)	7 (MF419443..56)
Hogsback 32°35′56.4”S 26°56′05.7″E	*R. dilectus*	11 (KU166490..500)	11 (MF419463..66)
Alice 32°48′55.2”S 26°50′21.5″E	*R. dilectus*	22 (KU166468..89)	*
East London 33°00′33.0”S 27°51′04.7″E	*R. dilectus*	14 (KU166645..58)	*
Fort Beaufort 32°43′19.8”S 26°37′31.5″E	*R. dilectus*	15 (KU166453..67)	*
Bethuli 30°29′02.3”S 25°56′03.5″E	*R. dilectus*	3 (KU166601..03)	*
Kaalplaas 25°35′28.0”S 28°09′26.4″E	*R. dilectus*	19 (KU166615..33)	5 (MF419577..86)
Rietvlei 25°52′20.7”S 28°16′38.6″E	*R. dilectus*	10 (KU166420..30)	10 (MF419625..32)
Windhoek 22°36′09.9”S 17°01′28.1″E	*R. bechuanae*	15 (KU166572..86)	11(MF419555..76)
Mariental 24°35′27.0”S 17°58′08.7″E	*R. bechuanae*	4 (KU166416..19	1 (MF419607..08)
Keetmanshoop 26°33′01.6”S 18°09′29.4″E	*R. bechuanae*	1 (KU166415)	*
Dronfield 28°44′36.7”S 24°48′52.1″E	*R. bechuanae*	23 (KU166501..23)	10 (MF419467..86)
Rooipoort 28°38′16.6”S 24°16′47.2″E	*R. bechuanae*	12 (KU166524..35)	*

* no sequences were available for these populations

Trapping and handling of animals and the collection of the ectoparasites is outlined in Engelbrecht et al. [6]. Ethical approval was obtained from the Stellenbosch University ethics committee (SU-ACUM11–00004) and permission for sampling rodents were obtained from local authorities and private land owners (Eastern Cape, CRO37/11CR; KZN wildlife OP4990/2010; Gauteng CPF 6–0153; CapeNature 0035-AAA007–00423; Northern Cape FAUNA 1076/2011, Free State 01/8091; Namibia 1198/2007). The taxonomic identification of all host specimens was genetically confirmed with sequencing [13, 15, 26].

### Molecular techniques

The genomic DNA of each parasite specimen was isolated using whole specimens and the Macherey-Nagel kit (GmbH & Co.) following the protocol of the manufacturer with slight modification (see [6] for more detail). Universal primers LCO1490 and HCO2198 [33], were used to amplify and sequence partial segments of the mitochondrial cytochrome oxidase subunit I (COI) gene. Cycle parameters were 1 min at 95 °C followed by a 10-cycle loop of 1 min at 95 °C, 45 °C and 72 °C, respectively. A 30-cycle loop followed using the exact same conditions as in the 10 cycle loop apart from increasing the 45 °C annealing temperature to 49 °C. All reactions were terminated by a final 5 min extension period at 72 °C. To amplify the nuclear intron Tropomyosin (TropoM) the TropoF5bis-F and TropoF5bis-R primers from Roy et al. [34] were used. The same cycle parameters as outlined above were used apart from first annealing at 49 °C for 10 cycles and this was followed by annealing at 54 °C for 30 cycles. All PCR reactions were conducted in 25 μl volumes and contained variable amounts of millipore water (pending on how much DNA was used), 3.5 μl of 25 mM MgCl_2_, 3 μl of 10X Mg^2+^-free buffer, 1.0 μl of a 10 mM dNTP solution and 1.0 μl (10 mM) of the respective primer pairs, 0.2 μl of *Taq* polymerase (5u/ul) and 2.5–4 μl of template DNA. PCR products were purified using a commercial kit (Macherey–Nagel, NucleoFast 96 PCR Kit). All cycle-sequencing reactions were performed using standard BigDye Chemistry and analysed on an automated sequencer (ABI 3730 XL DNA Analyzer, Applied Biosystems). Sequencing was only performed in both directions in instances where base calling was uncertain.

### Data scoring

To check the reliability and functionality of sequence reads, the BLASTN tool on GENBANK (NCBI BLAST) was used and all COI sequences were translated to amino acids with EMBOSS/TRANSEQ (EMBL – European Bioinformatics Institute). Sequences were edited and manually aligned using BIOEDIT ver. 7.0.9 [35]. The allelic phases of the TropoM nuclear gene were determined using PHASE ver. 2.1.1 [36, 37] as implemented in DNASP ver. 5.10.1 [38]. The PHASE analysis was performed for 100,000 generations with a burn-in of 10,000 generations. The analysis was considered resolved when probability values of 0.9 or higher were retrieved [36]. All subsequent analyses with the nuclear data were performed on the allelic data.

### Genetic diversity and phylogeographic analysis

The number of unique haplotypes (*h*), haplotype diversity (*H*_*d*_) and nucleotide diversity (π) were calculated for both gene fragments for all taxa using DNASP ver. 5.10.1 [38]. Population genetic differentiation (F_ST_) of parasite and host species were calculated using an analyses of molecular variance (AMOVA) in ARLEQUIN ver. 3.5.1.2 [39]. Statistical significance was assessed with 10,000 permutations. To establish the genetic structure among parasite haplotypes sampled at different localities, minimum spanning networks were constructed using PopART [40].

## Results

A total of 109 *L. muricola* individuals were characterized by 58 mtDNA haplotypes that translated into a haplotypic diversity of 0.97 (±0.00; Table 2). This measurement of diversity is considerably higher than what was found for the 182 *L. giganteus* individuals which were characterized by a mtDNA haplotypic diversity of 0.77 (±0.03; Table 2). At the nuclear DNA level, however, the two species showed virtually identical haplotypic diversity values (*L. muricola* = 0.98 (±0.01) vs. *L. giganteus* = 0.97 (±0.01); Table 2). However, when the mites occurring on the two *Rhabdomys* host species are considered separately, the mtDNA haplotypic diversity of the mites occurring on *R. bechuanae* are higher (mtDNA = 0.91 (±0.02); Table 2) when compared with *L. giganteus* occurring on *R. dilectus* (mtDNA = 0.62 (±0.00); Table 2). Nucleotide diversity values are also different between the two *Laelaps* species suggesting more similarity between haplotypes belonging to *L. muricola* (π = 1,5% (±0.00); Table 2) when compared to *L. giganteus* (π = 4,3% (±0.00); Table 2). These strong trends are, however, not visible when the nuclear data is compared and in fact show a trend that is rather opposite to the mtDNA data (Table 2). The haplotypic diversity of the rodent hosts used in this study range from a low of 0.72 (±0.05) in *M. natalensis* to a high of 0.95 (±0.02) in *R. dilectus* (Table 2). Nucleotide diversity was the lowest in *M. natalensis* (π = 1,5% (±0.00)) and the highest in *M. coucha* (π = 4,6% (±0.00); Table 2).

**Table 2:** Nuclear and mtDNA diversity estimates for the species used in this study

Species	N	bp	*h*	*H*_*d*_	π	Fst
*L. muricola* mtDNA	109	534	58	0.97 (±0.000)	0.015 (±0.001)	0.56 (*P* < 0.05)
*L. muricola* nuDNA	86	534	52	0.98 (±0.008)	0.026 (±0.002)	0.33 (*P* < 0.05)
*L. giganteus* mtDNA	228	644	58	0.77 (±0.03)	0.043 (±0.001)	0.87 (*P* < 0.05)
*L. giganteus* mtDNA on *R. dilectus*	173	644	36	0.62 (±0.001)	0.012 (±0.002)	0.53 (*P* < 0.05)
*L. giganteus* mtDNA on *R. bechaunae*	55	644	22	0.91 (±0.020)	0.018 (±0.001)	0.83 (*P* < 0.05)
*L. giganteus* nuDNA	182	534	79	0.972 (±0.005)	0.011 (±0.001)	0.48 (*P* < 0.05)
*L. giganteus* nuDNA on *R. dilectus*	138	534	65	0.961 (±0.008)	0.007 (±0.001)	0.12 (*P* < 0.05)
*L. giganteus* nuDNA on *R. bechaunae*	44	534	14	0.889 (±0.025)	0.006 (±0.001)	0.36 (*P* < 0.05)
*Rhabdomys* mtDNA	97	900	44	0.954 (±0.001)	0.060 (±0.001)	0.95 (*P* < 0.05)
*R. dilectus* mtDNA	43	900	26	0.944 (±0.023)	0.008 (±0.002)	0.18 (*P* < 0.05)
*R. bechuanae* mtDNA	54	900	18	0.887 (±0.028)	0.006 (±0.000)	0.79 (*P* < 0.05)
*M. coucha* mtDNA	91	545	24	0.849 (±0.029)	0.046 (±0.001)	0.38 (*P* < 0.05)
*M. natalensis* mtDNA	106	545	24	0.715 (±0.049)	0.003 (±0.000)	0.36 (*P* < 0.05)

The (N) number of individuals, number of base pairs analysed (bp), the number of unique haplotypes (*h*), haplotype diversity (*H*_*d*_) and nucleotide diversity (π) and global and F_st_ values are given for each comparison. Values in brackets represent the significance values or the standard deviations for mean estimates

The generalist *L. muricola* showed an overall low level of population differentiation between sampling localities at the mtDNA (F_st_ = 0.56, *P* < 0.05; Table 2) and nuclear DNA level (F_st_ = 0.33, *P* < 0.05; Table 2). This is in marked contrast with the species specialist *L. giganteus* where the level of population differentiation was much higher (mtDNA: F_st_ = 0.87, *P* < 0.05; nuDNA: F_st_ = 0.48, *P* < 0.05; Table 2). The level of population differentiation was highest between *R. bechuanae* hosts (F_st_ = 0.95, *P* < 0.05; Table 2) and lowest between populations of *M. natalensis* (F_st_ = 0.36, *P* < 0.05; Table 2).

**Fig. 2:**
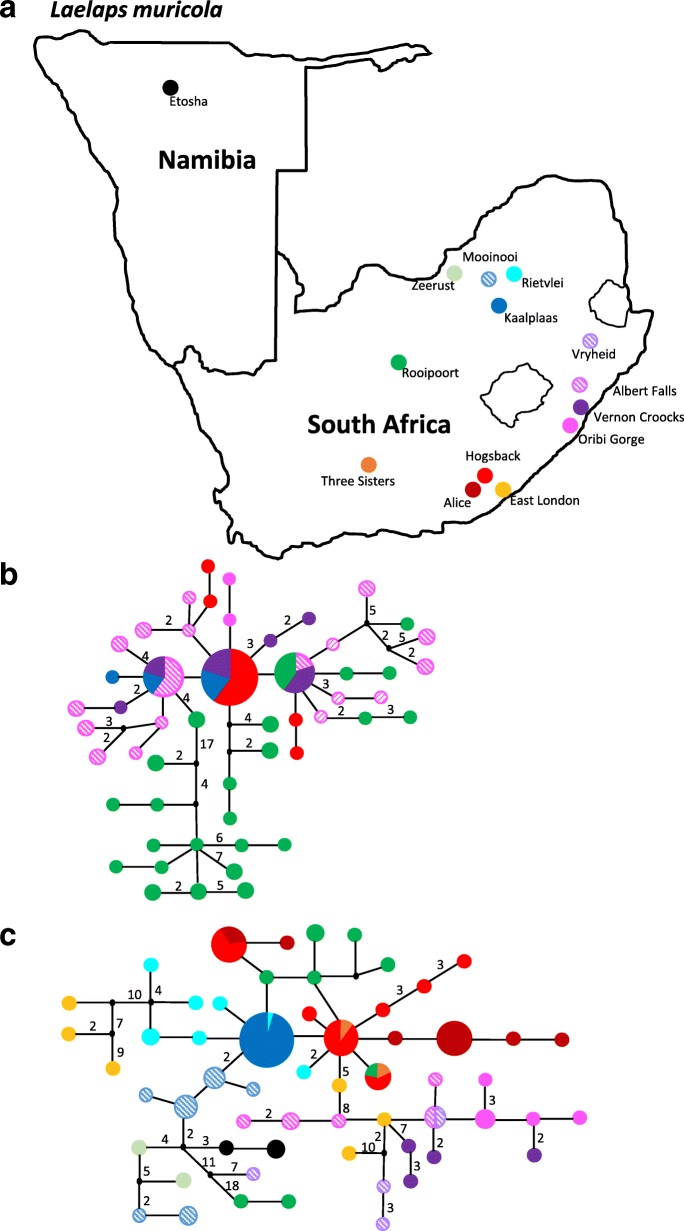
**a**) Sampling localities for *Laelaps muricola.* **b**) Nuclear TropoM and **c**) mtDNA COI haplotype networks. The haplotypes found are colour coded according to locality as per inset map. Haplotype sizes represent the frequency of the haplotypes and in instances where more than one site change separate haplotypes, the number of mutations are indicated along the braches. Haplotypes found at more than on locality are indicated by multiple colours in the same circle and correspond to the locality colouring in the inset

Minimum spanning network analyses based on the mtDNA COI data of *L. muricola* indicated 8.6% (5/58) shared haplotypes among localities while > 90% of the haplotypes are unique and locality specific (Fig. 2). The majority of unique haplotypes differed by singe site changes from each other but in some instances are markedly divergent and from the same locality (for example, Rooipoort has two haplotypes differing by at least 29 site changes from the central group, East London has four haplotypes differing by at least 10 steps from the central group and Vryheid has one haplotype differing by at least 18 steps from the central group; Fig. 2). At the nuclear DNA level, the same general patterns emerge where few haplotypes are shared among localities (three shared haplotypes in total), most haplotypes are closely related to each other, and some localities such as Rooipoort is characterized by very divergent lineages differing by as much as 17 mutational steps from the central network (Fig. 2). Although most localities are characterized by unique haplotypes, there is no clear geographic pattern present in the mtDNA or nuclear DNA haplotypes networks for *L. muricola* (Fig. 2)*.*

**Fig. 3:**
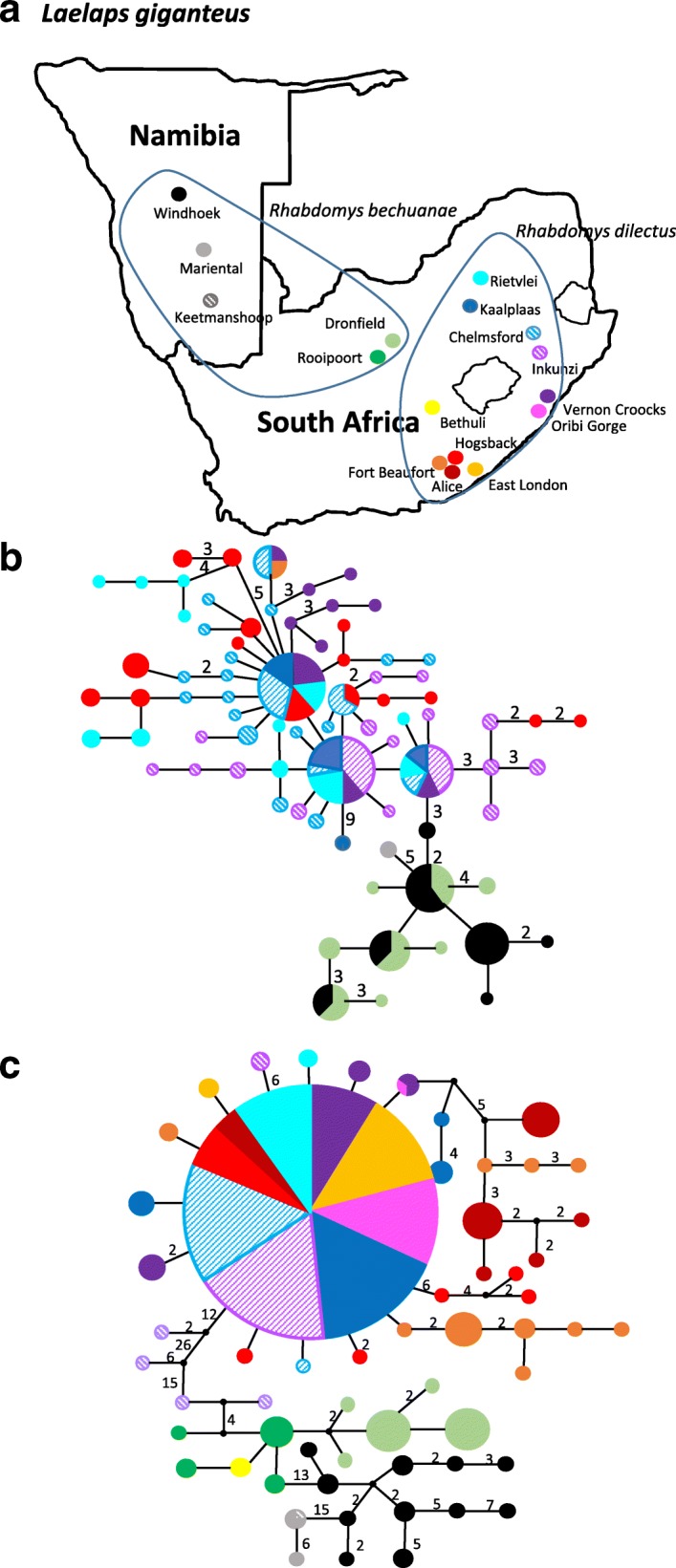
**a**) Sampling localities for *Laelaps muricola* and the approximate distribution of the two *Rhabdomys* host species demarkated by blue lines. **b**) Nuclear TropoM and **c**) mtDNA COI haplotype networks. The haplotypes found are colour coded according to locality as per inset map. Haplotype sizes represent the frequency of the haplotypes and in instances where more than one site change separate haplotypes, the number of mutations are indicated along the braches. Haplotypes found at more than on locality is indicated by multiple colours in the same circle and correspond to the locality colouring in the inset

The pattern of the species specialist, *L. giganteus* is markedly different. Two distinct mtDNA and nuclear DNA geographic assemblages can be identified (one confined to parasites sampled from *R. bechuanae* (with the exception of Bethuli) and one associated exclusively to *L. giganteus* sampled on *R. dilectus* (also see [13])*.* These intralineage patterns of the two genetic assemblages also differ from each other: the mtDNA haplotype network of parasites sampled from *R. bechuanae* is considerably structured based on sampling locality and haplotypes sampled at different localities generally differ by a large number of mutations (for example 13 site changes separate Windhoek from the Dronfield/ Rooipoort sampling site and 15 site changes separate the Windhoek sampling site from Mariental and Keetmanshoop; Fig. 3). In contrast, *Laelaps giganteus* sampled on *R. dilectus* show virtually no phylogeographic structure based on locality and in fact, 66% of the individuals (91/182 in total) share a single common haplotype (the shared haplotype is found at 9 of the 11 collection sites of *R. dilectus*)*.* The nuclear DNA haplotype network for *L. giganteus* is similarly less structured for the mites sampled from *R. dilectus* when compared to those sampled from *R. bechuanae* (Fig. 3)*.*

## Discussion

The phylogeographic structure obtained for the generalist *L. muricola* and the two specialists *L. giganteus* lineages provide new insights into mite dispersal, and more specifically also the effect of host movement and host range on the genetic diversity and dispersal of nest-bound ectoparasites. The large number of unique haplotypes found at each locality for both species support the notion of Engelbrecht et al. [13] that parasitic mites are intrinsicly restricted in their dispersal. This is mainly attributed to the fact that they spend a short time on the host when feeding, and thus often miss the boat (dispersal opportunity with the host, [41]). In addition, they have a 38% prevalence on the host (mean abundance of 1.54 (**±**0.19); [23]) and thus the few individuals that do disperse with the host drown on arrival (the effective population size of the newly colonized geographic population is too large to allow for new alleles to drift up in frequency, [41]).

Despite limited dispersal opportunities, marked differences in population structure were observed when the specialist *L. giganteus* (mtDNA F_st_ = 0.87, *P* < 0.05; NuDNA F_st_ = 0.48, *P* < 0.05) was compared to the generalist *L. muricola* (F_st_ = 0.56, *P* < 0.05; NuDNA F_st_ = 0.33, *P* < 0.05). Interestingly, however, the values for the mites are directly correlated to the level of mtDNA population differentiation of their respective hosts. *Rhabdomys* spp. (hosts for specialist mites) show a much higher average level of mtDNA population differentiation (F_st_ = 0.95, *P* < 0.05) when compared to *M. coucha* (most mobile hosts for the generalist mite) (F_st_ = 0.36, *P* < 0.05). Although the data here support the paradigm that parasite genetic structure depends on host dispersal [42], it is well documented that parasite life history [14, 43, 44] and abiotic factors [45] can equally influence parasite population differentiation. Since the majority of life history characteristics of the two *Laelaps* species used in our study are very similar [6, 23], and both species were most likely exposed to similar abiotic factors in their overlapping ranges (with eight identical sampling localities), we can most likely attribute the differences in population genetic structure of the parasites to either differences in life histories of the hosts, host dispersal ability, or species specificity of the parasite (or a combination of these three).

The differences in the dispersal patterns of the hosts of the two species specific *L. giganteus* lineages allows for more insights into the effect of host dispersal versus host range on parasite genetic structure. *Rhabdomys bechuanae* is most likely a group living rodent for most of the time and it has been suggested that the patchy distribution of the species is facilitated by the irregular spread of their food resources [46, 47]. The high level of geographic population differentiation of this rodent species is evident from their highly structured mtDNA haplotype network and consequently also a high level of population differentiation (F_st_ = 0.79, *P* < 0.05; Additional File 2: Figure S1). *Rhabdomys dilectus,* on the other hand, is more solitary and occurs in a more homogeneous habitat where resources are more evenly distributed and, as expected, this species have a less structured mtDNA haplotype network (Additional file 2: Figure S1) and shares more haplotypes among localities. The more homogeneous genetic pattern is also supported by the AMOVA analyses that indicated that 82% of the total mtDNA variation are intrapopulational and only 18% can be attributed to variation among sampling sites (F_st_ = 0.18, *P* < 0.05). Interestingly, when the levels of population differentiation of the two species specific *L. giganteus* lineages are compared, both the mtDNA and nuclear DNA indicated that the parasites occurring on *R. bechuanae* are highly structured (mtDNA F_st_ = 0.83, *P* < 0.05; nuDNA F_st_ = 0.36, *P* < 0.05) while those on *R. dilectus* show considerable lower levels of population structure (mtDNA F_st_ = 0.53, *P* < 0.05; nuDNA F_st_ = 0.12, *P* < 0.05). In light of the fact that both *L. giganteus* lineages are confined to single host species [13], these differences between the two species specific lineages are most likely linked to the dispersal abilities of their hosts and not to host specificity per se. Indeed, the specialist mite taxon occurring on hosts with a high dispersal potential (such as *L. giganteus* on *R. dilectus*; mtDNA F_st_ = 0.53, *P* < 0.05) show similar levels of population differentiation to a generalist parasite occurring on highly mobile hosts (such as *L. muricola* on *Mastomys*; mtDNA F_st_ = 0.56, *P* < 0.05)*.* In this particular comparison, host range contributes less while the ability of the host to move across the landscape contributes more to the population genetic structure of ectoparasitic mites.

Host range has also been implicated in affecting the intraspecific genetic diversity of ectoparasites [16]. The higher haplotypic diversity found for the generalist *L. muricola* (mtDNA *h* = 0.97 ± 0.00; nuDNA *h* = 0.98 ± 0.01), when compared to the specialist *L. giganteus* (mtDNA *h* = 0.77 ± 0.03; nuDNA *h* = 0.97 ± 0.01) fits the prediction that the specialist parasites are probably more influenced by local stochastic extinction events of their single hosts and will thus have a lower genetic diversity [16]. However, when the two specialist *L. giganteus* lineages were respectively compared for genetic diversity with the generalist *L. muricola* (mtDNA *h* = 0.97 ± 0.00; nuDNA *h* = 0.98 ± 0.01)*,* and interesting pattern emerged. When only the individuals occurring on *R. dilectus* are included in the analyses the support for lower genetic diversity on a generalist is stronger than before (mtDNA *h* = 0.62 ± 0.00; nuDNA *h* = 0.96 ± 0.01). The haplotypic diversity found for the specialist occurring on *R. bechuanae,* is much higher (mtDNA *h* = 0.91 ± 0.02; nuDNA *h* = 0.89 ± 0.03), and nearly approach similar values than that found for the generalist, *L. muricola.* In our case, the number of host species available to a parasite is thus also not always correlated with the intraspecific genetic diversity of the parasite (see [16]), and we propose that host factors are also important in this regard.

We attribute the differences in genetic diversity described above to differences in the evolutionary histories of the hosts. In the case of *L. muricola,* it has been proposed that both *Mastomys* species persisted in multiple refugia during paleoclimatic oscillations [15], and this could have contributed towards the retention of a large number of multiple unique locality specific haplotypes found in *L. muricola* (Fig. 2)*.* In addition, the generalist nature of *L. muricola* [6], and the high level of genetic diversity found in the third host, *Micaelamys* [25], provide further mechanisms to enhance high genetic diversity in *L. muricola.* In the case of *L. giganteus,* host evolution is equally important. *Rhabdomys dilectus* is confined to the mesic eastern area of southern Africa, a more continuous grassland region known to be subjected to repeated expansion and contraction cycles resulting from paleoclimatic changes [48, 49]. The lower genetic diversity and the large number of shared haplotypes found in the *L. giganteus* lineage occurring in the nests of the grassland *R. dilectus* (when compared to the *L. giganteus* lineage found on *R. bechuanae*), is most likely the result of multiple cycles of population fluctuations that co-incided with the palaeoclimatic expansion and contraction of C3 and C4 grasses [48, 49]. The higher genetic diversity, and also the highly structured pattern of locality specific haplotypes, found in *R. bechuanae* is more complex. It has been documented that from time to time, local populations of *Rhabdomys* occurring on the western dry area of southern Africa can experience severe fluctuations in population numbers due to factors such as severe droughts [47]. A large reduction in host availability may contribute to reductions in the genetic diversity of *L. giganteus* occurring on *R. bechuanae*, and in turn the patchiness of the suitable habitat for the host may facilitate structure among distant sampling sites. Some support for the latter is found in the fact that the populations of *R. bechuanae* is highly structured by locality throughout the range (Fig. 3).

## Conclusion

The outcome of the present study superficially support the SGVH hypothesis proposed by Li et al. [16]. When the genetic diversity of a generalist parasite is compared to a specialist, it was found, as predicted, to be lower in the specialist than in the generalist [19, 20]. When the amount of population genetic structure was compared it also fits the prediction that the specialist will show higher levels of population differentiation when compared to the generalist [17, 18]. However, the SGVH hypothesis at present considers host range as the driving factor in the equation. The findings of the present study emphasise that host dispersal and host evolution can play an even more important role in ectoparasite evolution. We thus propose a refinement of the SGVH hypothesis – “a species specialist *that is restricted by host dispersal* will show a higher level of population structure when compared to the generalist parasite and a specialist species are more prone to local stochastic extinction events than their more generalist counterparts, resulting in lower levels of genetic diversity in specialist when compared to more generalist parasites”.

## Additional files

Additional file 1: **Table S1.** Collection localities of host species included in this study together with the number of host sequences for the mtDNA COI gene fragment. Thirty new sequences were generated for this study indicated by * and the remainder were obtained from [15, 26]. Genbank accession numbers are given in each instance (DOCX 84 kb)

Additional file 2: **Figure S1.** a) Sampling localities of *Rhabdomys dilectus* and *R. bechuanae* individuals included in this study b) mtDNA COI haplotype network for *Rhabdomys dilectus* and *R. bechuanae* used in this study (PPTX 86 kb)
