# Supplementary material for: Comparative phylogeography of parasitic Laelaps mites contribute new insights into the specialist-generalist variation hypothesis (SGVH)
Source: BMC Evol Biol. 2018 Sep 3;18:131. doi: 10.1186/s12862-018-1245-7 (PMC6122474; doi:10.1186/s12862-018-1245-7)
Supplement: Supplementary file 1 — Table S1. Collection localities of host species included in this study together with the number of host sequences for the mtDNA COI gene fragment. Thirty new sequences were generated for this study indicated by * and the remainder were obtained from [15, 26]. Genbank accession numbers are given in each instance (DOCX 84 kb) [file 12862_2018_1245_MOESM1_ESM.docx]

Additional Table 1: Collection localities of host species included in this study together with the number of host sequences for the mtDNA COI gene fragment. Thirty new sequences were generated for this study indicated by * and the remainder were obtained from [15, 21]. Genbank accession numbers are given in each instance

| Host species | Locality | Parasite species | N of COI sequences  43 + 50 | Genbank Accession number |
| --- | --- | --- | --- | --- |
| *R. dilectus* |  |  |  |  |
|  | Fort Beaufort | *L. giganteus 1* | 9 | KC296585- KC296587; JQ003376 |
|  | Rietvlei | *L. giganteus 1* | *1 | MG561276 |
|  | Hogsback | *L. giganteus 1* | *3 | MG561278- MG561280 |
|  | Vryheid | *L. giganteus 1* | *1 | MG561281 |
|  | Oribi Gorge | *L. giganteus 1* | *2 | MG561282- MG561283 |
|  | East London | *L. giganteus 1* | *6 | MG561284- MG561289 |
|  | Inkunzi | *L. giganteus 1* | *3 | MG561290- MG561292 |
|  | Vernon Crooks | *L. giganteus 1* | *3 | MG561293- MG561295 |
|  | Kaalplaas | *L. giganteus 1* | *4 | MG561296- MG561299 |
|  | Bethuli | *L. giganteus 1* | *1 | MG561300 |
|  | Chelmesford | *L. giganteus 1* | 10 | KC296588- KC296592 |
| *R. bechuanae* |  |  |  |  |
|  | Dronfield | *L. giganteus 2* | *2 | MG561301- MG561302 |
|  | Keetmanshoop | *L. giganteus 2* | 12 | JQ003302, JQ003303; KC296594-KC296600 |
|  | Mariental | *L. giganteus 2* | *4 | JQ003323- JQ003325 |
|  | Windhoek | *L. giganteus 2* | 17 | JQ003466-JQ003470 |
|  | Rooipoort | *L. giganteus 2* | 15 | JQ003453 |
| *M. natalensis* (106) |  |  |  |  |
|  | East London | *L. muricola* | 6 | KJ466191 |
|  | Albert Falls | *L. muricola* | 15 | KJ466183; KJ466192; KJ466193 |
|  | Chelmsford | *L. muricola* | 3 | KJ466190 |
|  | Inkunzi | *L. muricola* | 4 | KJ466189 |
|  | Vryheid | *L. muricola* | 4 | KJ466184; KJ466194; KJ466195 |
|  | Kavango | *L. muricola* | 15 | KJ466185-KJ466188 |
|  | Lusaka | *L. muricola* | 15 | AB752648.1; AB752630.1; AB752587.1; AB752578.1; AB752657.1; AB752544.1; AB752627.1; AB752619.1; AB752611.1; AB752598.1; AB752588.1; AB752575.1; AB752572.1; AB752561.1; AB752550.1 |
|  | Banamaiya | *L. muricola* | 15 | AB752760.2; AB752754.1; AB752758.2; AB752763.2; AB752746.1; AB752755.1; AB752753.1; AB752752.1; AB752739.1; AB752737.1; AB752735.1; AB752733.1; AB752731.1; AB752718.1; AB752757.2 |
|  | Kabulamwanda | *L. muricola* | 9 | AB752683.1; AB752677.1; AB752681.1; AB752675.1; AB752674.1; AB752704.1; AB752702.1; AB752765.1; AB752671.1 |
|  | Katoshi | *L. muricola* | 6 | AB752668.1; AB752667.1; AB752669.1; AB752662.1; AB752660.1; AB752658.1 |
|  | Kantengwa | *L. muricola* | 10 | AB752699.1; AB752695.1; AB752693.1; AB752689.1; AB752775.1; AB752684.1; AB752685.1; AB752633.1; AB752774.1; AB752773.1 |
|  | Leopard Hill | *L. muricola* | 2 | AB752581.1; AB752583.1 |
|  | Maala | *L. muricola* | 2 | AB752714.1; AB752712.1 |
| *M. coucha* |  |  |  |  |
|  | Grootfontein | *L. muricola* | 13 | KJ466152; KJ466153; KJ466154; KJ466155; KJ466156; KJ466157; KJ466158; |
|  | Mariental | *L. muricola* | 6 | KJ466180; KJ466181 |
|  | Otjiwarongo | *L. muricola* | 7 | KJ466170; KJ466171; KJ466172; KJ466173; KJ466174 |
|  | Okahandja NAM | *L. muricola* | 14 | KJ466159; KJ466160 |
|  | Uniab River | *L. muricola* | 1 | JQ667766.1 |
|  | Waterberg Plateau | *L. muricola* | 2 | JQ667768.1; JQ667769.1 |
|  | Omatjene Research | *L. muricola* | 1 | JQ667770.1 |
|  | Windhoek NAM | *L. muricola* | 1 | JQ667771.1 |
|  | Rietvlei SA | *L. muricola* | 15 | KJ466161; KJ466162; KJ466163; KJ466164; KJ466165 |
|  | Chelmsford Nature | *L. muricola* | 1 | KJ466182 |
|  | Bloemfontein | *L. muricola* | 1 | JQ667767.1 |
|  | Kaalplaas SA | *L. muricola* | 14 | KJ466175; KJ466176; KJ466177; KJ466178; KJ466179 |
|  | Mooinooi SA | *L. muricola* | 15 | KJ466166; KJ466167; KJ466168; KJ466169 |
