# Supplementary material for: Comparative phylogeography of parasitic Laelaps mites contribute new insights into the specialist-generalist variation hypothesis (SGVH)
Source: BMC Evol Biol. 2018 Sep 3;18:131. doi: 10.1186/s12862-018-1245-7 (PMC6122474; doi:10.1186/s12862-018-1245-7)
Supplement: Supplementary file 2 — Figure S1. a) Sampling localities of Rhabdomys dilectus and R. bechuanae individuals included in this study b) mtDNA COI haplotype network for Rhabdomys dilectus and R. bechuanae used in this study (PPTX 86 kb) [file 12862_2018_1245_MOESM2_ESM.pptx]

## Slide 1
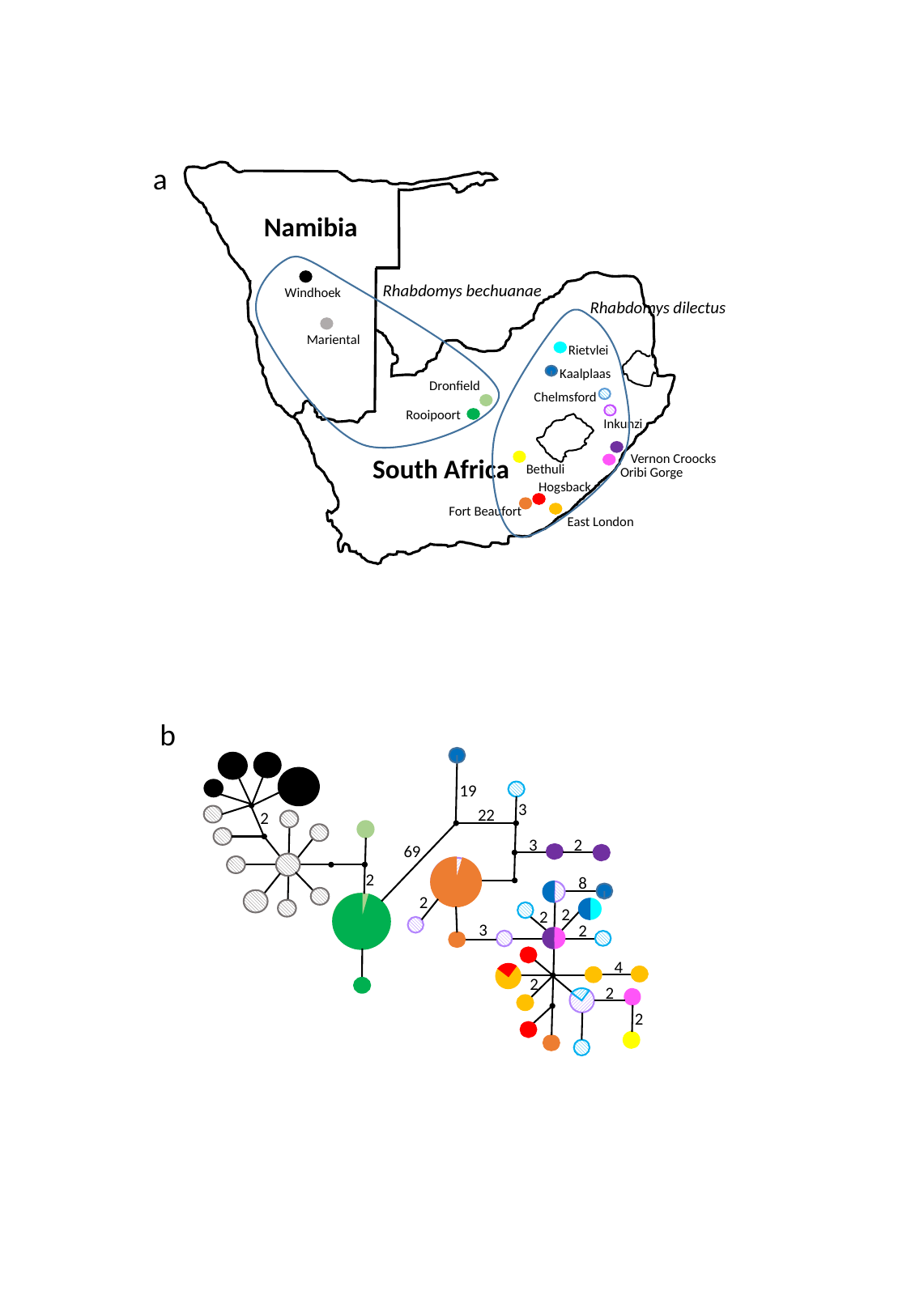

a
Namibia
Windhoek
Mariental
Rietvlei
Kaalplaas
Dronfield
Chelmsford
Rooipoort
Inkunzi
Vernon Croocks
South Africa
Bethuli
Oribi Gorge
Hogsback
Fort Beaufort
East London
Rhabdomys bechuanae
Rhabdomys dilectus
b
19
3
22
2
3
2
69
2
8
2
2
2
3
2
4
2
2
2
